# Paralysis of the Hand and Forearm from Smoking

**Published:** 1866-05

**Authors:** A. J. H. Banks

**Affiliations:** Evercreech, Somersetshire


### Paralysis of the Hand and Forearm from Smoking.

By Dr. A. J. H. Banks, Evercreech, Somersetshire.

[Dr. Banks considers that impaired nervous energy and even actual paralysis produced by the excessive use of tobacco are much more common than is usually supposed. All or most will probably endorse the assertion of impaired nervous energy resulting from excessive smoking, but cases of actual paralysis resulting from this cause must be very rare. We never remember seeing one which we could without any doubt assign to this cause.]

In February last I was consulted by P. S——. He was forty-eight years of age, tall, well made, and of rather spare figure. I found him suffering from what he called "dropped hand." It had lasted for some considerable time, and he had been under treatment for it; but finding it get worse, had discontinued for some weeks before applying to me. Recently, owing to a good deal of numbness and uncomfortable sensation down the right side and right lower extremity, he began to fear he should be unable to persue his work (which was that of gate-keeper on the Somerset and Dorset line), and this induced him to apply to me. I ascertained that he was an inveterate smoker, and, supposing this to be the cause of his ailment, I advised him to refrain from the habit altogether. I then gave him an ordinary purgative, placed blisters on the back and front of the arm and forearm, dressed these with an ointment composed of one grain of strychnine and one ounce of spermaceti ointment, and subsequently gave him one-sixteenth of a grain of strychnine in a pill three times daily. In ten days he had perfect use of the limb, lost all his disagreeable symptoms, and has been at work regularly since that time without any signs of it returning. He tells me that he thought, and that the surgeon who was attending him was of the same opinion, that the loss of power arose from local external injury; and that it had been treated by cold lotions and wet bandages, assiduously applied, under which it became gradually worse. I think this mistake is not unfrequently made.—*Lancet, Sept.* 3, 1864, *p.* 280.

